# Preoperative exercise in patients undergoing total knee arthroplasty: a pilot randomized controlled trial

**DOI:** 10.1186/s40945-020-00085-9

**Published:** 2020-08-05

**Authors:** Pascale Gränicher, Thomas Stöggl, Sandro F. Fucentese, Rolf Adelsberger, Jaap Swanenburg

**Affiliations:** 1 The University Center for Prevention and Sports Medicine (UCePS), Balgrist University Hospital, Forchstrasse 319, 8008 Zurich, Switzerland; 2 Department of Epidemiology, CAPHRI School for Public Health and Primary Care, Maastricht University, Peter Debyeplein 1, 6229 HA Maastricht, The Netherlands; 3 Department of Sport and Exercise Science, University of Salzburg, Schlossallee 49, 5400 Hallein/Rif, Austria; 4 Department of Orthopaedic Surgery, Balgrist University Hospital, University of Zurich, Forchstrasse 340, 8008 Zurich, Switzerland; 5 Wearable Computing Lab Zurich, ETH Zurich, Gloriastrasse 35, 8092 Zurich, Switzerland; 6 Integrative Spinal Research ISR, Department of Chiropractic Medicine, Balgrist University Hospital, Forchstrasse 340, 8008 Zurich, Switzerland

**Keywords:** Prehabilitation, Knee osteoarthritis, Preoperative physical therapy

## Abstract

**Background:**

The purpose of this study was to assess the effect of preoperative physiotherapy (PT) on functional, subjective and socio-economic parameters after total knee arthroplasty (TKA).

**Methods:**

20 patients (mean ± SD: age 67 ± 7 years) scheduled for TKA at Balgrist University Hospital between July 2016 and March 2017 were randomly assigned to a control (CG) or intervention (IG) group. 3 to 4 weeks prior to surgery the IG completed 5 to 9 sessions of PT containing proprioceptive neuromuscular facilitation (PNF) techniques, endurance training and individually indicated interventions. Measurements were executed at baseline, preoperative and 3 months after TKA. The primary outcome measure was the Stair Climbing Test (SCT), secondary outcome measures were the knee range of motion (ROM) and the level of physical activity using Lysholm Score (LS) and Tegner Activity Scale (TAS). The subjective and socio-economic parameters were the Patients’ Global Impression of Change (PGIC) scale, inpatient rehabilitation time, preoperative pain levels and metabolic equivalent (MET), postoperative intake of analgesics and overall costs.

**Results:**

No difference between IG and CG was found for SCT (F (2/36) = 0.016, *p* = 0.984, η2 = 0.004). An interaction between group and time was shown for TAS (F (18/1) = 13.890) with an increase in the IG (*p* = 0.002, η2 = 0.536). The sub-item “pain” within the LS presented a higher pain-level in CG (F (18/1) = 4.490, *p* = 0.048, η2 = 0.974), while IG showed a higher preoperative MET compared to CG (*p* = 0.035). There were no other significant changes. The CG produced 21.4% higher overall costs, took more analgesics and showed higher preoperative pain levels than the IG.

**Conclusions:**

Findings show that preoperative therapy improved the level of physical activity before and after TKA and resulted in a clinically relevant gain in TAS.

**Trial registration:**

ClinicalTrials.gov Identifier; NCT03160534. Registered 19 May 2017

## Highlights

- Prehabilitation improves levels of physical activity before and after total knee arthroplasty
- Clinically relevant benefits on Tegner Activity Scale after prehabilitation
- Tendencies for shorter inpatient rehabilitation after preoperative exercise

## Background

Total knee arthroplasty (TKA) is the elective surgical procedure after failing conservative management in patients suffering from advanced knee osteoarthrosis (KOA) [45]. Often, severe KOA is accompanied by constant pain, restricted joint flexibility, weakness of the quadriceps muscle and reduced knee functionality in sports and activities of daily living (ADLs) [24, 37]. Although pain levels and joint flexibility are to be improved after surgery, 20 to 30% are not satisfied with the result [7, 38]. These patients do not achieve significant symptomatic enhancement or their impairments in ADLs become even worse [21]. In their review, Canovas and Dagneaux [7] highlighted the ability of walking down the stairs as an important factor contributing to patients’ satisfaction after TKA. This goal may be compromised, because a great proportion does not achieve comparable strength to healthy persons 2 years after TKA [34].

During preoperative waiting time, not only joint flexibility and pain levels get worse, but joint-surrounding muscles further atrophy due to reduced facilitation and neuromuscular inhibition [14]. As a result, perceived participation in ADLs and the level of physical activity (PA) deteriorate [32].

Several systematic reviews and meta-analyses showed that physiotherapeutic prehabilitation may reduce postoperative limitations in ADLs and improve functional outcomes after TKA [15, 42, 44, 46]. Promising training interventions accompanied by patient education are endurance training to improve overall fitness [1, 11, 32] and proprioceptive neuromuscular facilitation (PNF) techniques to increase joint ROM, improve neuromuscular performance [17, 39] and reduce pain levels [9, 31, 35]. But the lack of specific information in previously tested prehabilitation protocols concerning exercises, dosage and intensity makes it difficult to draw a conclusion [32, 44]. There is no consent, which dosage should be applied in preoperative exercise therapy. It is a tightrope walk to apply the ideal individual training intensity and not provoking an exacerbation of pain symptoms at the same time in patients awaiting TKA [15, 32, 44].

In a timeframe of 3 to 4 weeks, it is realistic to improve overall cardiovascular capacity and neuromuscular performance in joint surrounding muscles [3] as well as influence pain levels in order to enhance functional performance in ADLs [28].

Preoperative levels of PA in patients awaiting TKA correlate with postoperative outcomes, even years afterwards [25, 32]. Therefore, the aim of this study is to improve functional ability of the knee by training neuromuscular performance of the quadriceps and hamstring muscles and enhancing joint flexibility as well as aerobic capacity. Besides the functional benefits, the prehabilitation protocol applied within this study intends to improve subjective and socio-economic parameters such as preoperative pain levels, postoperative intake of painkillers, the length of stay at an inpatient rehabilitation facility and overall costs.

## Methods

### Participants

Eligible patients older than 18 years receiving unilateral TKA were asked to participate in the study and recruited over a period of 9 months (July 2016–March 2017) at the Department of Orthopaedics of Balgrist University Hospital. Patients were excluded from participation if they suffered from muscle weakness due to secondary neurological diagnosis, high Body Mass Index (BMI > 33.0 kg/m^2^), depression, patellar instability, patella alta (Caton Deschamps Index > 1.2) or if they showed signs of inflammation such as swelling, warming, acute pain or redness apart from lack of function, or a planned osteotomy of tibial tuberosity. No payment or compensation was given to the study participants.

### Design

This is a pilot randomized controlled trial with patients being randomly assigned to an intervention group (IG) or control group (CG). All procedures followed the Helsinki Declaration and all participants provided written informed consent. The ethics committee of the Canton of Zurich approved the study under BASEC 2016–00258. ClinicalTrials.gov Identifier; NCT03160534. All measurements and therapy sessions were conducted at the Balgrist University Hospital, Zurich, Switzerland.

### Intervention

The aim of the preoperative training intervention was to increase the level of PA in ADLs (e.g. walking up and down the stairs) while correcting evasive movements, improve neuromuscular coordination of the joint surrounding muscles and prevent relieving postures during these activities. The maintenance of a higher preoperative level of PA due to better joint flexibility, improved intramuscular coordination of the thigh muscles and increased overall aerobic capacity was aspired [49]. The training consisted of 5 to 9 sessions of physiotherapy within 3 to 4 weeks before surgery, with the following content: 1) 10-45 min endurance training on a bicycle ergometer, pedal trainer, treadmill, or crosstrainer (patients’ choice) with light to moderate exercise intensity (40–70% of maximum heart rate) without pain provocation; 2) PNF techniques of quadriceps and hamstring muscles known as the contract-relax-antagonist-contract (CRAC) form and conducted with the assistance of a physiotherapist [17, 39]; 3) patient education: patients were comprehensively informed during therapy sessions regarding the topics of self-training at home, pain management and coping strategies, answering questions concerning pre- and postsurgical procedures and joint-friendly, physiological movement patterns; 4) individual interventions when indicated, e.g. strengthening exercises [22], sensori-motor training (e.g. integration of proprioceptive inputs on balance and gait) [13, 30, 48] or electromyostimulation training [20]. The strengthening exercises were performed underloaded (coordination at 10–20% of 1-Repetition Maximum (1RM)) or at submaximal intensity (strength endurance at 30–50% of 1RM and hypertrophy at 50–80% of 1RM) and targeted muscles of the lower extremities (e.g. quadriceps, hamstrings, calves, abductors). The sensori-motor training consisted of balance exercises on one leg (3–4 sets à 30–60s), sessions on how to walk with crouches and training of physiological movement patterns in ADLs. Whereas, the CG was asked to keep their activity level the same as before the baseline measurement and not to start a new type of therapy or training during the preoperative stage. The intensity during every intervention was set as high as manageable without pain provocation.

### Assessments

Outcomes were assessed on three measurement events; at baseline three to 4 weeks before surgery, immediately before surgery and at follow-up 3 months post-surgery. Following the baseline assessment, all patients were instructed to keep a personal diary during the three to 4 weeks prior to surgery as well as during the 3 months post-surgery period.

### Primary outcome

The Stair Climbing Test (SCT) measures the time used to ascend and descend a flight of eight 16 cm high steps with a depth of 30 cm [12]. Patients were asked to complete the test at usual walking speed, feeling safe and comfortable. Light use of the handrail or assistive devices were allowed but not encouraged if the patient felt unsafe. The handrail was to be used only for guidance and not for pulling. Completion of a trial was determined when both feet arrived on the final stair. The time to negotiate the stairs was measured by a stopwatch, as this method shows excellent test-retest intercorrelations (0.93) [36] in SCT. The minimal detectable change is set at 0.102 s [23]. A practice trial was completed and the mean of three subsequent trials was used for analysis. A 30-s break was held between the practice trial and each of the following trials.

### Secondary outcomes

The range of motion (ROM) of the involved knee was assessed by a standard goniometer (clear plastic, 18 cm of length) in supine position, where the patient was asked to slide the heel towards the buttock as close as possible. The examination of knee ROM in patients with KOA has adequate reliability with an ICC coefficient of 0.96 for flexion and 0.81 for extension [10] and the minimal detectable change lies at 7.9° for flexion and 3.8° for extension [29]. The Lysholm Score (LS) was used for assessment of patients’ perception of knee function and activity level in ADLs [4, 40]. Scores were categorized from “poor” (64) to “excellent” (95–100), with a score of 100 being considered as symptom-free [40]. The item “pain” was analyzed separately in order to retrieve information about the change of pain levels before and after surgery. The Tegner Activity Scale (TAS) was applied for assessment of work and sporting activities. The patients indicated the highest level of participation that best described their current level of activity. A score of 0 represents “sick leave or disability pension because of knee problems” where a score of 10 stands for “competitive sports such as football or alpine skiing (national or international level) [40]. The German versions of the LS (Lysholm-G) and the TAS (Tegner-G) were validated for patients after TKA [40]. They showed acceptable psychometric performances for the Lysholm-G and the Tegner-G scales as outcome measures for patients after TKA. The smallest detectable change for the TAS equals 1.4 points and 1 point for the LS [47].

To determine the perceived level of change after surgery, the Patient Global Impression of Change (PGIC) questionnaire was assessed at follow-up. The patients self-rated on the scale from 1 “very much improved” to 7 “very much worse” [18]. It has been shown that the PGIC is valuable in evaluating physiotherapeutic outcomes, as it requires little time and therefore may be ideal for the clinical setting [41]. Information concerning the postoperative risk of discharge to a rehabilitation facility and length of stay at an inpatient rehabilitation was extracted from the clinic’s information system.

### Subjective and socio-economic outcomes

In their personal diary, all patients answered standardized questions about their preoperative pain level (daily), documented their daily activities in order to calculate their individual metabolic equivalent (MET) and listed total costs (e.g. preoperative therapies, walking aids, medication) prior to surgery.

After surgery, patients had to document the amount and costs of postoperative medication and post-acute care services (e.g. payment for home health agencies) as well as the amount of rehabilitative ambulant physiotherapy sessions, in their diary until the follow-up measurement. Data regarding the amount of preoperative therapy sessions and prescribed length of stay at an inpatient rehabilitation facility was retrieved from the clinics information system in order to calculate the complete treatment costs.

### Randomization and blinding

Allocation to the IG or CG followed a computer-generated randomization list. The group assignments were concealed in envelopes. The assignments were revealed after baseline testing by a secretary who was independent of the study. The recruiting investigator was unaware of the next participant’s allocation and therefore blinded at baseline measurement. The same physiotherapist conducted all measurements and 81 out of 85 preoperative interventions, substituted by one other physiotherapist in case of absence. Both therapists followed a previously instructed, standardized intervention protocol. Preoperative and follow-up measurements were not blinded, as the same physiotherapist who carried out the preoperative interventions, acted as investigator. All three measurements were supervised by an independent and blinded collaborator in order to ensure objectivity. Patients were not replaced after dropout or withdrawal.

### Data analysis

All data were checked for normality using the Shapiro Wilk test. Two-way ANOVA with repeated measures (2 groups, 3 time points) was applied for comparison of SCT, knee ROM, LS and TAS between the two groups and the three time points. Additionally, effect size Eta-squared (η^2^) was calculated (< 0.02 = trivial; 0.02 to 0.13 = small; 0.13 to 0.26 = medium; > 0.26 = large). For the PGIC, clinically significant improvement was defined as very much or much improved condition (score 1 or 2) [41]. To determine significant group differences between the lengths of stay at inpatient rehabilitation facilities, mean values of stationary weeks were compared between the two groups by independent t-test. Level of significance was set at α = 0.05 for all comparisons.

The additional subjective and socio-economic outcomes were extracted from the personal diaries. Independent sample t-tests were used to compare the groups by 1) preoperative pain levels, 2) preoperative MET, 3) postoperative pain medication and 4) total costs. All statistical procedures were performed with IBM SPSS 23 statistical software package (SPSS Inc., Chicago, Illinois, USA).

## Results

### Participant flow

**Fig. 1:**
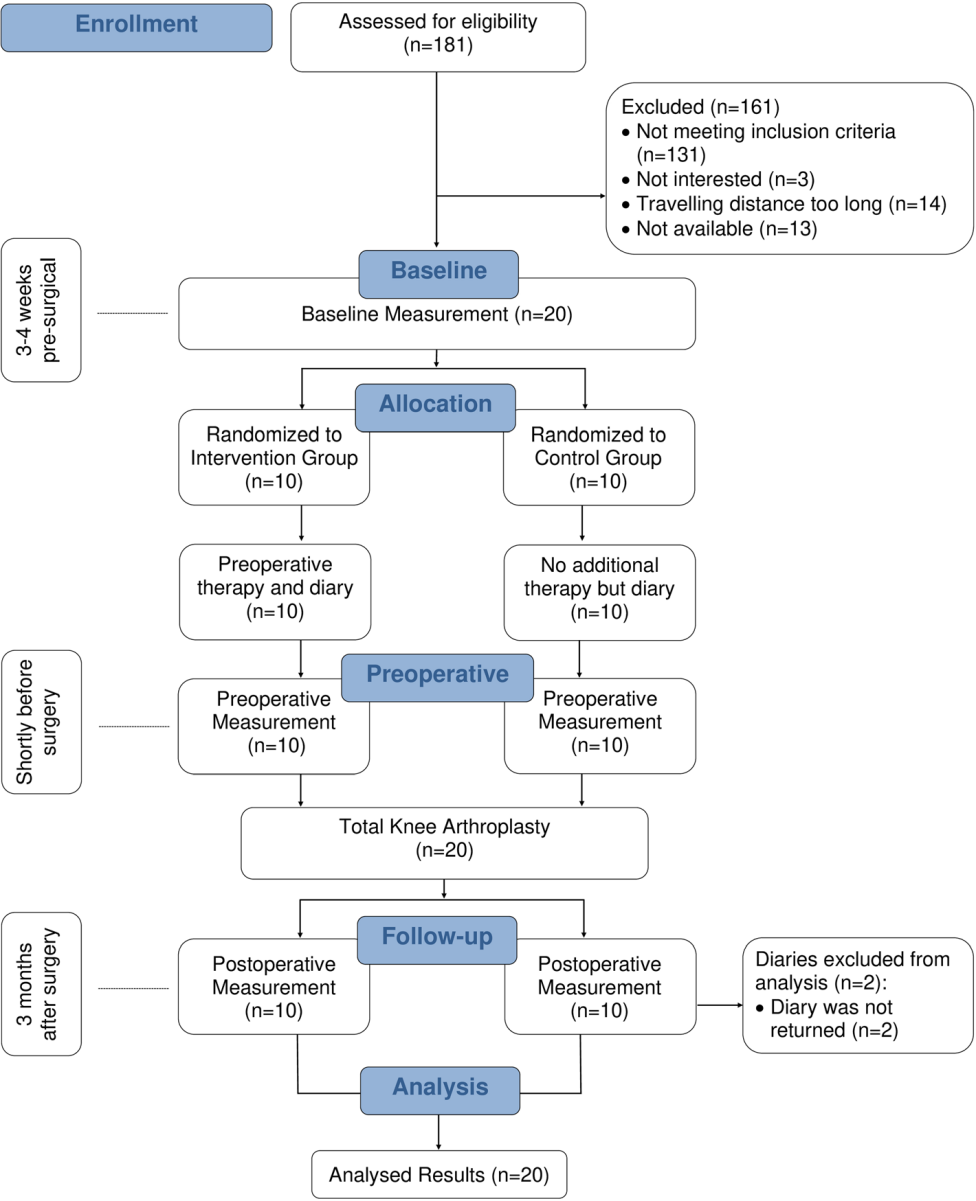
The graph shows the flowchart of the TKA patients for the intervention study

The flow of patients through the study is shown in Fig. 1.

### Characteristics

Patients’ characteristics are displayed in Table 1. There were more males in the IG, but no significant differences were observed between the groups at baseline. All patients suffered from severe KOA (Kellgren and Lawrence Grade 4) where the conservative approach has been exploited and indication for TKA given by the orthopedist.

**Table 1:** Baseline characteristics of all TKA patients and those in the intervention and control group

	Intervention Group (*n* = 10)	Control Group (*n* = 10)	All patients (*n* = 20)	*p*
	Mean (SD) Min-Max	Mean (SD) Min-Max	Mean (SD) Min-Max	
Male	7	5	12	n/a
Female	3	5	8	n/a
Age (years)	66.6 (7.52) 53–80	68.1 (7.68) 55–81	67.35 (7.44) 53–81	0.664
Height (cm)	174.2 (11.17) 150–185	168.35 (6.59) 155–178	171.28 (9.42) 150.0–185.0	0.171
Weight (kg)	90.2 (19.378) 65–116	80.3 (14.21) 56–103	85.25 (17.29) 56–116	0.209
Deviation of the knee joint axis (°)	9.0 (3.95) 3–14.5	12.4 (4.24) 6–19	10.7 (4.35) 3–19	0.809

*Abbreviation*: *SD* Standard deviation

*n* = Sample size

*p* = Independent t-test

n/a = not applicable

*significant *p* < 0.05

### Adherence to the intervention program

Two patients were not able to complete the full set of nine sessions due to absence (vacation, work). Overall exercise compliance was 96.7% regarding completion of diaries but there were no dropouts as all patients finished the study and no adverse events were reported within the study. Table 2 shows individual interventions of the IG during preoperative period.

**Table 2:** Individual interventions in IG and CG

Patient-Nr.	Main problem / restriction (subj./obj.)	Prehabilitation	Total knee arthroplasty
01	• Pain while walking or standing	• None.	• Medacta GMK MyKnee, LBS Sphere, Inlay 10 mm fixed bearing medial pivot
02	• Pain during knee loading	• None.	• Zimmer Persona, Inlay 10 mm fixed bearing PS
03	• Reduced knee extension • Pain	• PNF stretching: hamstrings, quadriceps • Endurance: cycling ergometer • Patient education: pain management, self-mobilization	• Medacta GMK MyKnee, Inlay 10 mm fixed bearing PS
04	• Pain during and after walking	• None.	• Medacta GMK MyKnee, Inlay 10 mm fixed bearing PS
05	• Knee varus • Non-physiological gait pattern Reduced quadriceps innervation	• PNF stretching: quadriceps • Endurance: cycling ergometer • Patient education: knee mechanics and movement patterns • Electromyostimulation training training: quadriceps • Sensori-motor training: gait training without walking stick, balance exercises, movement pattern	• LINK Gemini SL, PorEx, Inlay 10 mm fixed bearing PS
06	• Pain	• PNF stretching: hamstrings, quadriceps • Endurance: cycling ergometer • Patient education: pain management, self-training • Sensori-motor training: balance, gait • Strength training: coordination	• Medacta GMK MyKnee Hinge, Inlay 10 mm fixed bearing Hinge
07	• Pain when walking up the stairs or more than 500 m	• None.	• Medacta GMK MyKnee, Inlay 14 mm fixed bearing PS
08	• Pain when walking down the stairs.	• None.	• Medacta GMK MyKnee, Inlay 10 mm fixed bearing PS
09	• Impaired gardening • Pain after long walks	• None.	• Medacta GMK MyKnee, Inlay 14 mm fixed bearing PS
10	• Pain after resting for more than 15 min	• None.	• Medacta GMK MyKnee Revision, Inlay 10 mm fixed bearing SC
11	• Pain • Reduced performance at work	• PNF stretching: hamstrings, quadriceps • Endurance: walking on treadmill, cycling ergometer • Patient education: pain management, self-training, movement patterns • Electromyostimulation training: quadriceps • Sensori-motor training: balance, gait • Strength training: coordination, strength endurance, hypertrophy	• Zimmer Persona, Inlay 10 mm fixed bearing PS
12	• Reduced knee flexion • Slight knee pain	• PNF stretching: quadriceps • Endurance training: cycling ergometer, aqua jogging • Patient education: pain management, self-training /−mobilization • Sensori-motor training: balance, gait	• Medacta GMK MyKnee, Inlay 12 mm fixed bearing PS
13	• Reduced knee flexion and extension • Pain • Impaired sport performance	• PNF stretching: quadriceps and hamstrings • Endurance training: cycling ergometer • Patient education: intensity strength training, self-mobilization • Sensori-motor training: gait, balance, movement pattern • Strength training: coordination, strength endurance, hypertrophy	• Zimmer Persona, Inlay 10 mm fixed bearing PS
14	• Knee valgus • Slight pain • Reduced ADL performance	• PNF stretching: quadriceps, hamstrings • Endurance training: crosstrainer • Patient education: self-management, physiological movement pattern • Proprioceptive training: gait, balance, ADL • Strength training: coordination, strength endurance	• Medacta GMK MyKnee LBS Sphere, Inlay 13 mm fixed bearing medial pivot
15	• Weakness • Reduced knee flexion and extension • Slight pain	• PNF stretching: quadriceps, hamstrings • Endurance training: pedal trainer • Patient education: self-training, self-mobilization • Strength training: coordination, strength endurance	• Medacta GMK MyKnee, Inlay 10 mm fixed bearing PS
16	• Pain in the morning and after walking down the stairs	• None.	• Zimmer Persona, Inlay 10 mm fixed bearing PS
17	• Stiffness • Pain after walking	• None.	• Medacta GMK MyKnee LBS Sphere, Inlay 13 mm fixed bearing medial pivot
18	• Pain when knee is loaded.	• None.	• Zimmer Persona, Inlay 11 mm fixed bearing PS
19	• Knee varus, reduced knee flexion, impaired sport performance	• PNF stretching: quadriceps, hamstrings • Endurance training: cycling ergometer • Patient education: self-training /−mobilization, movement patterns, training intensity • Sensori-motor training: gait, balance • Strength training: coordination, strength endurance, hypertrophy	• Medacta GMK MyKnee LBS Sphere, Inlay 10 mm fixed bearing medial pivot
20	• Impaired 18-hole golf performance due to pain	• PNF stretching: quadriceps, hamstrings • Endurance training: cycling ergometer, treadmill walking • Patient education: self-training/−mobilization, movement patterns, training intensity • Sensori-motor training: gait, balance • Strength training: coordination, strength endurance	• Medacta GMK MyKnee Revision, Inlay 10 mm fixed bearing semiconstrained

Intensity strength training: coordination = 10–20% of 1 RM underloaded and not performed to momentary muscle failure; strength endurance = 30–50% of 1 RM performed to momentary muscle failure; hypertrophy = 50–80% of 1 RM performed to momentary muscle failure

### Effects

No effects between the IG and CG were demonstrated for primary outcomes in SCT (F = 0.252, *p* = 0.621, η^2^ = 0.014).

The secondary outcomes revealed no change between the IG and CG in knee ROM (F = 0.350, *p* = 0.561, η^2^ = 0.087), and LS (F = 1.877, *p* = 0.188, η^2^ = 0.253), whereas the separate analysis of the pain-level as a sub-item of the LS showed a significant difference between IG and CG (F = 4.490, *p* = 0.048, η^2^ = 0.974).

The TAS displayed an increase over time and group difference as well as a large effect size (F = 13.890, *p* = 0.002, η^2^ = 0.941). There were no group differences in the PGIC (*p* = 0.307) and the length of stay at a rehabilitation facility (*p* = 0.486). Results are shown in Table 3.

**Table 3:** Results of primary and secondary outcomes on each assessed time point in both groups, changes (*n* = 10 in each group)

Measure	Baseline		t-test	Preoperative		Postoperative	
	IG	CG		IG	CG	IG	CG
	Mean (SD) Min-Max	Mean (SD) Min-Max	*p*	Mean (SD) Min-Max	Mean (SD) Min-Max	Mean (SD) Min-Max	Mean (SD) Min-Max
Stair Climbing Test (sec)	12.37 (3.74) 6.58–18.26	13.54 (7.35) 6.14–27.33	0.658	12.68 (5.00) 5.60–23.58	14.11 (9.19) 5.71–34.87	12.58 (4.64) 6.70–23.23	13.59 (5.30) 5.13–24.02
ROM (°)	113.30 (16.99) 90–135	120.60 (15.43) 80–135	0.328	116.70 (13.24) 95–135	116.20 (17.39) 70–134	100.50 (18.65) 60–120	103.5 (13.69) 90–128
Lysholm Score (all items)	53.50 (21.59) 26–90	59.00 (13.59) 41–84	0.504	70.70 (20.21) 30–97	54.80 (18.52) 13–83	87.10 (9.04) 72–100	69.11 (14.87) 52–96
Lysholm Score (only pain)	10.50 (7.25) 0–20	9.50 (4.38) 5–15	0.713	13.00 (10.06) 0–25	9.00 (6.15) 0–20	25.00 (0.00) 25–25	16.50 (9.14) 5–25
Tegner Activity Scale	2.50 (0.71) 1–3	2.10 (0.738) 1–3	0.232	3.10 (0.568) 2–4	2.00 (0.816) 1–3	3.80 (0.789) 3–5	2.50 (0.850) 1–4
PGIC			0.307			2.50 (2.17) 1–7	2.10 (1.45) 1–4
Length of stay in rehabilitation facility (weeks)			0.486			1.0 (1.49) 0–4	1.5 (1.64) 0–4

*Abbreviations*: *IG* Intervention Group, *CG* Control Group, *ROM* Range of motion, *PGIC* Patients Global Impression of Change, *SD* Standard deviation

Values given as Mean (SD) or range = Min-Max

*p* = Independent t-test

*significant *p* < 0.05

### Subjective and socio-economic outcomes

There were beneficial effects within the IG as the calculated MET displays. When excluding intervention-related activities, the IG was significantly more active during the preoperative phase compared to the CG (*p* = 0.035). Results are shown in Table 4.

**Table 4:** Summary of socio-economic outcomes in both groups measured at different time intervals

Measure	IG	CG	*p*	Ratio IG: CG
	Mean / Min-Max	Mean / Min-Max		
Preoperative pain level (NRS)	4.27 / 0–8	4.53 / 0–8	0.524	1: 1.06
Change of pain level (NRS) from baseline to preoperative assessment	1.5 / 0–8	0.38 / 0–4	0.189	4: 1
Preoperative MET	982.4 / 0–3033	438.2 / 0–4181	0.035*	1.7: 1
Postoperative medication (number of months)	1.9 / 1–3	2.4 / 1–3	0.150	0.79: 1
Pre- and rehabilitation costs per patient (CHF)	5272.8	6704.4	0.730	0.79: 1

*Abbreviations*: *IG* Intervention Group, *CG* Control Group, *NRS* Numeric Rating Scale, *MET* Metabolic Equivalent, *SD* Standard deviation

Values given as Mean (SD) or range = Min-Max

*p* = Independent t-test

*significant *p* < 0.05

After the intervention period, pain levels measured from baseline to preoperative assessment improved in the IG by 30%, while the CG showed an improvement of 6.5% on NRS (*p* = 0.189). The evaluation revealed that the intake of painkillers was 21% higher in the CG compared to IG after surgery. The IG had an average length of intake of 1.9 months, whereas the CG was on medication for 2.4 months (*p* = 0.150).

Comparing the costs and amount of prehabilitation and rehabilitation; preoperative therapy sessions, postoperative stationary rehabilitation and therapies were calculated for each group. As Table 5 shows, the total costs of pre- and postoperative therapy and stationary rehabilitation were 21.3% higher in the CG (CHF 6704) compared to the IG (CHF 5273) (*p* = 0.730) [19, 33].

**Table 5:** Summary of average total costs of pre- and postoperative therapies per patient

Costs per patient	IG (CHF)	CG (CHF)
Preoperative PT	467	0
Postoperative PT	1161	1223
In-patient rehabilitation	3645	5481
Total costs per patient (CHF)	5273	6704

*Abbreviations*: *PT* Physical therapy, *IG* Intervention Group, *CG* Control Group

## Discussion

Changes during the intervention phase were not due to different preliminary settings, as homogeneity at baseline for both groups was demonstrated (see Table 3). A tendency in SCT indicated that the IG could maintain its level of mobility and functional performance, while the CG rapidly degraded towards the time of operation. This observation matches the findings of McKay et al. [28] and Fortin et al. [16]: Conditions of patients suffering from KOA become worse the closer surgery gets and prolonged waiting time has a negative effect on physical performance [14]. As Baker and McKeon [2] point out, patients with severe KOA also show negative alterations in climbing up and down the stairs before TKA.

By training and preserving physiological movement pattern and therefore functional performance of the knee using PNF techniques, postoperative transmission back to normal ADL activities seemed to be easier. Whereas the ability to climb stairs showed tendencies of improvement in the IG, knee ROM experienced a similar retrogression in both groups 3 months after surgery compared to the baseline assessment. As no maximum knee ROM is required to climb the stairs, a decreasing knee flexion did not necessarily influence the preoperative SCT.

In terms of perceived functional performance and participation in ADLs after TKA, the results in LS and TAS suggest faster recovery regarding knee stiffness, joint mobility and pain in the IG and indicate a significant influence of prehabilitaton on postoperative TAS outcome measures. The significant difference of preoperative MET may be an explanation, as the patients in IG were more active before, and therefore also after surgery [25]. Similar results showed the study by Clode et al. [11]: After an eight-week exercise and education program, patients’ pain and level of PA in ADL improved prior to TKA. The patients felt well prepared and therefore, postsurgical satisfaction was positively influenced by the program. The minimal clinically relevant change for the TAS equals 1.4 points [47], which is nearly exceeded by comparing the ratings at follow-up measurements in both groups in the present study but is below the value after considering changes from baseline to follow-up assessment. The IG improved almost up to level 4, which indicates an active lifestyle [28] and implies a significant enhancement in quality of life [43]. Due to the small sample size, single outliers strain distinct results and make it difficult to draw conclusive statements from these questionnaires. Nevertheless, it may be important to consider the IG’s development of a more active lifestyle as high ratings in TAS prior to surgery [43] and preoperative MET suggested when drawing conclusions to perceived outcome after surgery. Whether the reason for these results is a better intramuscular coordination due to PNF techniques, individual exercises or improved aerobic capacity is not fully clear.

The findings of the meta-analysis by Chen et al. [8] who observed a significantly reduced rate of inpatient rehabilitation after preoperative physiotherapy match the difference in length of stay between IG and CG presented in this study. It has to be taken into account that in Switzerland, the decision for or against inpatient rehabilitation does not exclusively depend on the medical condition after surgery but, among other things; e.g. on the insurance policy. After the four-week prehabilitation phase, the case costs added by postoperative inpatient rehabilitation were 33% higher in the CG compared to the IG, mainly due to inpatient care. An extended prehabilitation period may shorten the length of stay at the hospital and therefore reduce overall case costs even more, as Calatyud et al. [6] managed with an eight-week, high-intensity training and Rooks et al. [37] achieved by applying a six-week intervention program prior to TKA.

The advantageous results of preoperative pain levels documented in the patients diaries and revealed by the item “pain” in the LS in IG compared with CG can be explained by the findings of Lewit and Simons [26]: Their outcome showed an immediate pain relief as well as a long-lasting pain reduction after PNF techniques. The current results support these findings as not only the IG’s pain levels within the LS improved between baseline and preoperative assessment, but the effect was transferred to the 3 months follow-up where this group reported no pain at all. Baker and McKeon [2] as well as Canovas and Dagneaux [7] emphasize this statement by pointing out the importance of preoperative pain as a relevant prognostic factor in postoperative outcome and quality of life after TKA. A similar correlation was found for preoperative level of PA and postoperative outcome.

A higher preoperative MET predicts a better functional result, as Scott et al. [38] showed in their work. A possible connection may be drawn to the shorter time in inpatient rehabilitation facilities documented by the IG in this study, as its patients were significantly more active before surgery compared to the CG. There is a tendency for a higher presurgical level of PA and a lower pain score to predict faster recovery, including reduced pain medication, as findings of Baker and McKeon [2] confirm.

Individual estimation of societal participation defined by the scores in TAS provided valuable evidence supporting preoperative physiotherapy. Prehabilitation improves pre- and postoperative level of PA in ADLs. This may affect perceptions directly by giving the patients more insight and control over their medical care and therefore reporting higher physical functioning at follow-up measurement [5]. As lower fear-avoidance beliefs correlate with lower pain levels and better performance [27], patient education prior to TKA adds up to functional benefits. Further, the diverse expectations and demands of patients receiving a TKA show that today’s health system is facing multi-layered challenges. Decreasing financial resources, and at the same time, increasing claims for health services in function- and participation-related areas are making the situation more acute [32]. The clinical focus has moved away from implant survival to patient-reported outcomes. The focus of scientific evaluation begins to concentrate on a patient’s experience and level of satisfaction after TKA and not only on objective outcome parameters [1, 21].

### Interference factors and limitations

The study we present here was a pilot study and the number of participants therefore low. Nevertheless, patient compliance was high, with 96.7% compared with other studies reporting 75–90% [46].

The strong point of this study was its randomized controlled design. But, although randomization process and baseline assessment were blinded, the same physiotherapist performed all three measurements as well as the preoperative intervention program. In that context, the prehabilitation program was also conducted in one single clinic and the same surgical team undertook all operations. As postoperative care was individual and took place in various private practices and clinics, there was no possibility to verify the reported data on the amount of therapy sessions. Therefore, the calculation for the total amount of costs depended partly on subjective reports of the patients.

## Conclusion

The results of this pilot randomized controlled trial indicate that presurgical physiotherapy likely has a small effect on SCT, knee ROM, LS and the length of stay at a rehabilitation facility. The IG increased its level of PA by almost 2 points on the TAS after TKA, where recreational sport is possible again. The effect in the IG was strong when compared with the CG, suggesting evidence that preoperative intervention enhances the level of PA in ADL prior and after TKA.

The significant improvements after prehabilitation presented in TAS and the sub-item “pain” within the LS as well as in preoperative MET emphasize the trend for accelerated recovery and improved patients’ perceived outcome by optimizing prognostic parameters prior to TKA.

## Data Availability

The datasets used and/or analyzed during the current study are available from the corresponding author on reasonable request.

## Abbrevations

1RM: 1-Repetition maximum
ADLs: Activities of daily living
BMI: Body Mass Index
CG: Control group
CRAC: Contract-relax antagonist-contract
IG: Intervention group
KOA: Knee osteoarthritis
LS: Lysholm Score
MET: Metabolic equivalent
η^2^: Eta-squared
PA: Physical activity
PGIC: Patients’ Global Impression of Change
PNF: Proprioceptive neuromuscular facilitation
PT: Physiotherapy
ROM: Range of motion
SCT: Stair Climbing Test
TAS: Tegner Activity Scale
TKA: Total knee arthroplasty
